# Relapsing paradoxical reaction in miliary tuberculosis: a case report and literature review

**DOI:** 10.1002/rcr2.658

**Published:** 2020-09-09

**Authors:** Abdullah Mobeireek, Nasser A. Al Shekail

**Affiliations:** ^1^ Section of Pulmonary Medicine, Department of Medicine King Faisal Specialist Hospital and Research Centre Riyadh Saudi Arabia; ^2^ Department of Medicine Ibri Hospital Ibri Oman

**Keywords:** Corticosteroids, immune reconstitution, paradoxical reaction, tuberculosis

## Abstract

Paradoxical reaction (PR) after initiating anti‐tuberculous therapy (ATT) is a well‐recognized immune phenomenon. Less recognized, however, is a pulmonary reaction that is associated with miliary tuberculosis (TB), which can be a source of diagnostic confusion and progress to respiratory failure and acute respiratory distress syndrome (ARDS). We report an elderly patient who developed PR associated with respiratory failure following ATT for miliary TB, with radiological and pathological documentation. He responded to corticosteroids, but relapsed twice when the dose was reduced. It is imperative to be familiar with this form of PR to avoid diagnostic pitfalls and initiate appropriate therapy.

## Graphical abstract

Worsening of miliary tuberculosis after initiation of anti‐tuberculous therapy is uncommon but is potentially serious and can cause diagnostic confusion. It is, therefore, prudent to recognize this phenomenon and manage it appropriately with corticosteroid therapy.

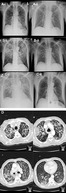

## Introduction

Tuberculosis (TB) is a common infection both worldwide and locally with significant morbidity and mortality as well as an economic burden [1]. Currently, different regimens of anti‐tuberculous therapy (ATT) are widely available that are quite effective in limiting the infection to a great extent. Occasionally, however, adverse drug reaction may be encountered during the course of the treatment. Among these is an interesting phenomenon, a paradoxical reaction (PR) which occurs following ATT. A “PR” is defined as a transient clinical or radiological worsening of pre‐existing tuberculous lesions or the development of new lesions in a patient who initially improves with anti‐TB therapy [2]. Reports of PR in extra‐pulmonary sites such as the peripheral lymph nodes or intracranial tuberculomas are plentiful [3, 4]. However, pulmonary involvement with PR is much less common, but may cause diagnostic confusion and can progress to life‐threatening respiratory failure [5]. Here, we describe an unusual case of an elderly patient with miliary TB who developed PR that was associated with respiratory failure, and in whom corticosteroids led to significant response. Furthermore, the patient developed two subsequent relapses after lowering the dosage of corticosteroids.

## Case Report

**Figure 1:**
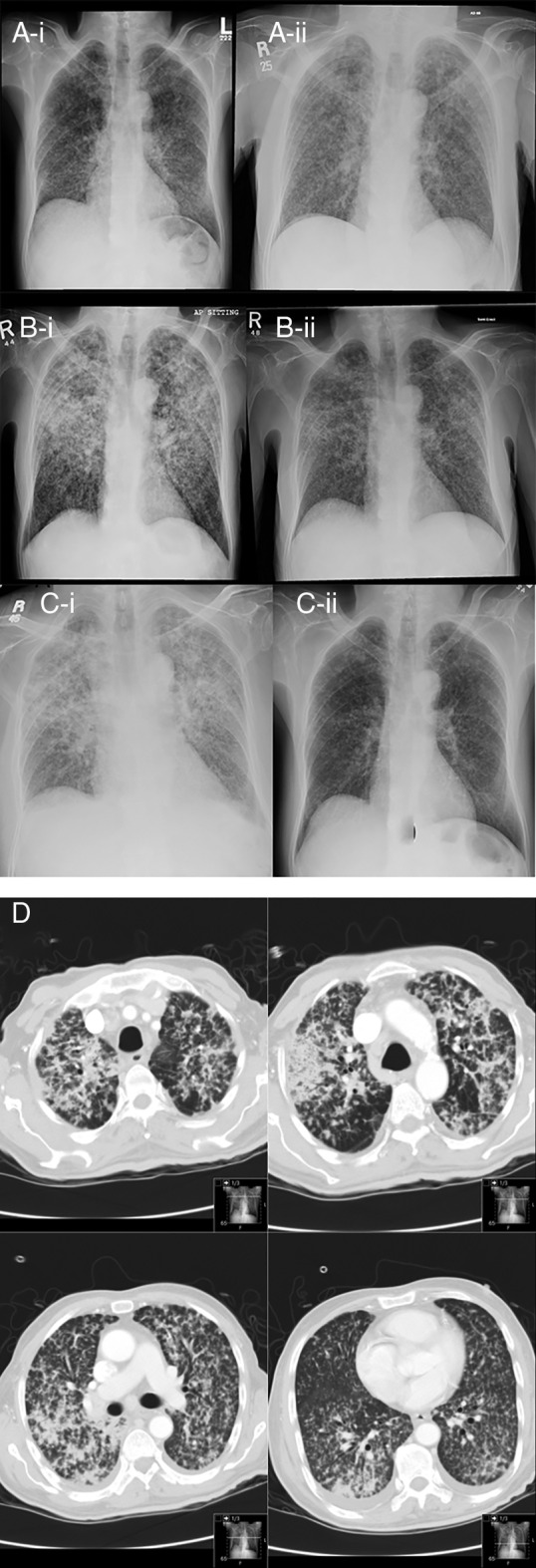
Chest radiographs on the first (A), second (B), and third admissions (C) (i, on admission; ii, on discharge). (D) Selected images of computed tomography (CT) of the chest during the second admission.

A 76‐year‐old gentleman presented with generalized fatigability, anorexia, weight loss, and fever for four weeks prior to presentation. He was known to have diabetes mellitus on oral hypoglycaemics and prior treatment of transitional cell carcinoma of the urinary bladder three years before presentation with no recurrence on follow‐up. On examination, the patient appeared cachectic and was febrile. Chest examination revealed bilateral fine crepitations. The rest of the examination was unremarkable. The chest radiograph showed diffuse bilateral miliary opacities (Fig. 1A‐i). Laboratory tests showed increased inflammatory markers (erythrocyte sedimentation rate (ESR): 43 and C‐reactive protein (CRP): 94). Complete blood count, renal, hepatic functions, and electrolytes were within normal limits. The tuberculin test was negative and three sputa for acid‐fast bacilli (AFB) were also negative. Bronchoalveolar lavage (BAL) was negative for AFB and malignant cells. The transbronchial biopsy (TTB) showed poorly formed granuloma. The patient was started on four first‐line ATT on suspicion of miliary TB (isoniazid 300 mg daily, rifampin 600 mg daily, ethambutol 1000 mg daily, and pyrazinamide 1250 mg daily). In addition, because of his constitutional symptoms, he was started on prednisone 20 mg daily. The patient was in hospital for three weeks and showed gradual clinical improvement, although his chest radiograph, as expected, did not change significantly (Fig. 1A‐ii). He was discharged with the same ATT and prednisone to be tapered gradually over two weeks.

When seen in the clinic after four weeks, the patient's symptoms recurred despite adherence to ATT. Chest radiograph showed significant progression of the disease and computed tomography (CT) of the chest showed diffuse miliary nodules and new air space consolidations and interval development of mediastinal lymphadenopathy (Fig. 1B‐i, D). Again, sputum and BAL were negative for AFB, bacteria, and fungi, including *Pneumocystis jirovecii*. Also, cytology of BAL did not show evidence of viral cytopathy, and TBB showed organizing pneumonia and no pathogens. The working diagnoses included failure of therapy, drug‐resistant TB, a bacterial co‐infection, and a PR. To cover the possibility of drug‐resistant TB and drug‐related fever, his ATT was modified to include second‐line ATT drugs (ciprofloxacin 500 mg twice daily and amikacin 750 mg every other day, and in addition, ethambutol was continued) while awaiting results of mycobacterial cultures. Subsequently, sputa and previous BAL cultures were all negative for mycobacteria, but the DNA amplification test was positive for Mycobacterium Tuberculosis. As he was persistently febrile, a trial to hold his ATT was performed and drugs were restarted sequentially to exclude a drug‐related reaction. However, the patient remained febrile and hypoxaemic. Thereafter, a PR was thought to be most likely and he was started on prednisone 40 mg daily. The patient showed marked clinical improvement in his constitutional symptoms and came off oxygen, as well as significant radiological resolution (Fig. 1B‐ii). He was discharged on 40 mg daily prednisone tapering dose, in addition to his original ATT regimen.

**Figure 2:**
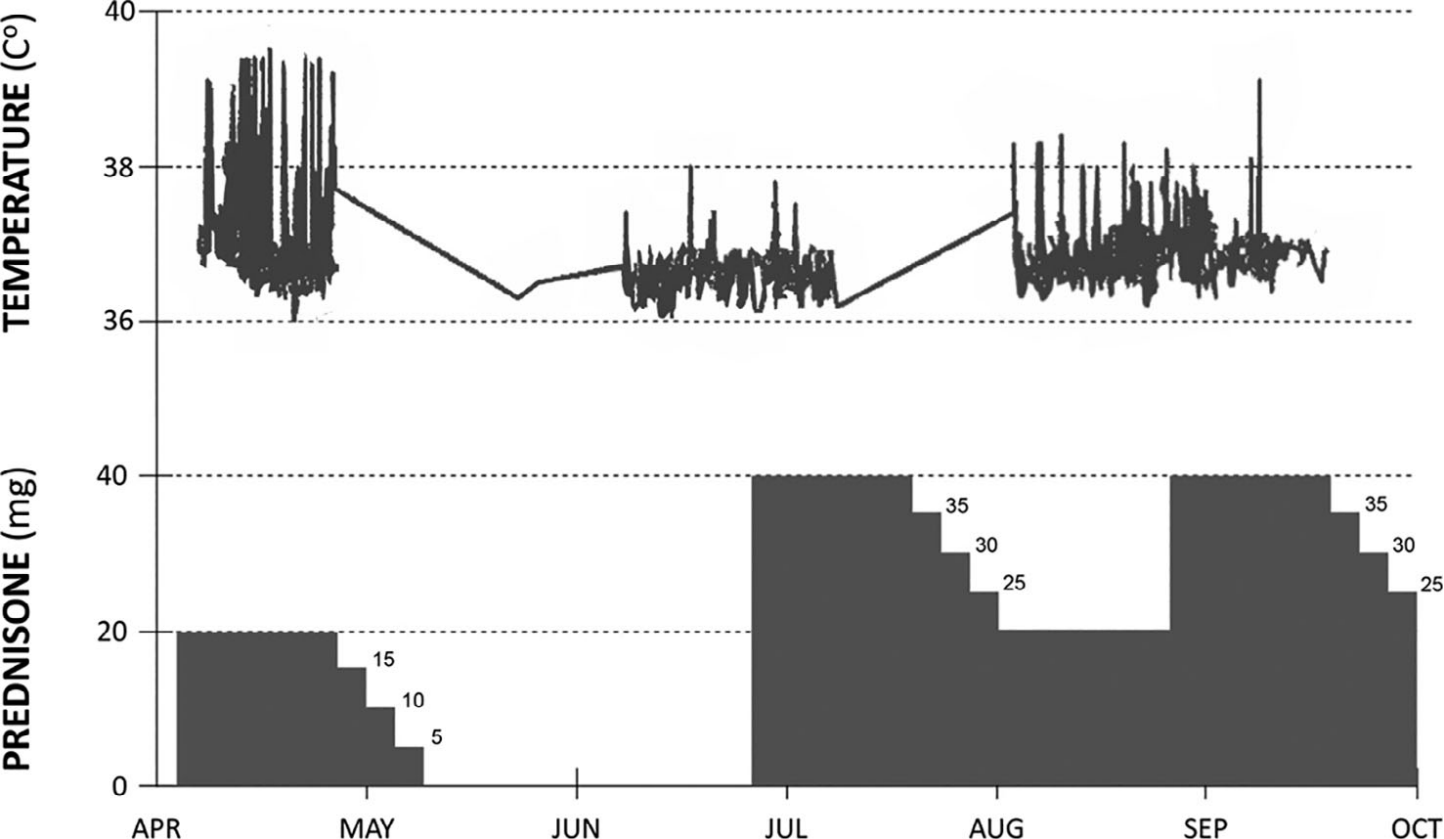
Temperature pattern in the three admissions related to the dose of prednisone.

Three weeks later, the patient came back to the emergency department complaining of fever. Again, adherence to ATT was ensured and at that point he was on 20 mg prednisone daily. Clinically, he was febrile, tachycardic, and tachypnoeic, and his oxygen saturation was 93% on 4 L/min of oxygen. His chest examination showed bilateral crepitations. The chest radiograph (Fig. 1C‐i) showed slight worsening with acute‐on‐chronic changes compared with the last one prior to discharge. An increase in white cell count and inflammatory markers was again noted. Sputum smear was positive again for DNA amplification. The possibility of a PR was entertained and prednisone doubled to 20 mg twice daily. The patient improved symptomatically and clinically, and his temperature returned to normal. He was discharged after improvement with isoniazid (INH), rifampicin, and prednisone. ATT was continued for total of 12 months. Prednisone was tapered slowly and discontinued after eight weeks. Figure 2 shows temperature pattern in the three admissions related to the dose of prednisone. He remained well and had no further relapses after a follow‐up of 17 months and the last chest radiograph is shown in Figure 1C‐ii.

## Discussion

The incidence of PR after the initiation of ATT has been variable depending on the site of the TB infection, but a large study with mixed forms of TB found an incidence of 2.4% [6]. Reports of PR date back to 1955 [7], with a renewed interest later during the HIV epidemic, when it was observed in HIV‐positive patients on initiation of anti‐retroviral therapy [8]. PR is believed to occur as a result of recovery of the immune system with an enhanced inflammatory response, hence it was also termed “immune reconstitution syndrome.” It has been postulated that PR results from the influx of both type 1 and 2 helper lymphocytes following the destruction of the TB bacilli, followed by release of a variety of cytokines that lead to an exaggerated immune response [5, 9]. We could not find pathological description of PR in previous reports. In our case, lung biopsy showed non‐specific organizing pneumonia and resolution of the previously noted granuloma suggesting response to ATT.

PRs were reported in multiple organs, including lymph nodes, brain, and skin, but pulmonary involvement is rare. Even more rare is its occurrence with miliary TB; only 29 cases have been reported in the literature so far in non‐HIV‐infected patients [8]. In such situation, respiratory failure may develop and evolve to ARDS, often with poor outcome [5]. In a patient with a recent diagnosis of TB, PR may cause diagnostic confusion, which was the case with our patient. Other possibilities such as therapy failure, poor compliance, drug resistance, and co‐infection were entertained. A thorough investigation of these possibilities was performed, and at one point his conventional ATT was modified to cover the possibility of drug resistance. It is essential to exclude these potential causes so that appropriate anti‐inflammatory therapy can be implemented rapidly. Our patient developed respiratory failure and it is possible that corticosteroids halted the progression to full blown ARDS. Interestingly, he developed two subsequent relapses that responded to an increase in dosage, which emphasizes the need for close follow‐up and careful tapering of corticosteroids.

Corticosteroids are the only anti‐inflammatory drugs that have been used in the management of PR to the best of our knowledge. They are safe and effective provided the sensitivity of the mycobacterial strain is known and reconciled with ATT, which should not be discontinued during the management of PR [10]. Furthermore, if rifampicin is included in the regimen, the dose of the corticosteroid may need adjustment as it accelerates the metabolism [10]. This may explain why our patient developed a third relapse despite being on 20 mg of prednisone. With advancement in the knowledge of mechanisms of PR, specific biologic therapies might have a role in the future for severe PR with better efficacy and safety profiles.

In conclusion, PR can present with worsening clinical status following the initiation of ATT and cause diagnostic confusion. Clinicians need to suspect and confirm the PR by excluding other possible causes. Close follow‐up is also necessary as relapses can occur. More research is warranted into the pathophysiological mechanisms of PR and the role of newer more specific anti‐inflammatory therapies in its management.

### Disclosure Statement

Appropriate written informed consent was obtained for publication of this case report and accompanying images.
